# Case Report: Fatal Necrotizing Pneumonia by Exfoliative Toxin *etE2*-Producing *Staphylococcus aureus* Belonging to MLST ST152 in The Netherlands

**DOI:** 10.3390/microorganisms13071618

**Published:** 2025-07-09

**Authors:** Wouter J. van Steen, Monika A. Fliss, Ethel Metz, Klaus Filoda, Charlotte H. S. B. van den Berg, Bhanu Sinha, Erik Bathoorn

**Affiliations:** 1 Department of Intensive Care, University Medical Center Groningen, University of Groningen, 9713 GZ Groningen, The Netherlands; 2 Department of Medical Microbiology and Infection Prevention, University Medical Center Groningen, University of Groningen, 9713 GZ Groningen, The Netherlandsb.sinha@umcg.nl (B.S.); 3 Department of Intensive Care, Ommelander Ziekenhuis Groningen, 9679 BJ Scheemda, The Netherlands

**Keywords:** *Staphylococcus aureus*, PVL, exfoliative toxin, molecular epidemiology, surveillance

## Abstract

We present a case of fatal necrotizing *Staphylococcus aureus* pneumonia with underlying influenza A (H3) infection. Next-generation-sequencing-based analysis revealed that the *S. aureus* isolate harbored the newly recognized exfoliative toxin *etE2* gene. Molecular epidemiologic analysis showed that the isolate belonged to the MSSA ST152 lineage, harboring PVL genes and *edinB* co-located to *etE2* as distinctive virulence factors. The *etE2* gene is present in all isolates of this lineage co-located to the exotoxin gene *edinB*, both implicated in the destruction of tissue integrity. We alert as to the global emergence of this lineage causing serious infections in patients.

## 1. Introduction

This study illustrates the case of a previously healthy young adult who was hospitalized in the Netherlands for the treatment of a community-acquired necrotizing *Staphylococcus aureus* (MSSA) pneumonia with an underlying influenza A (H3) infection. Necrotizing pneumonia is a disease entity with a high mortality rate that is characterized by a rapidly deteriorating clinical course, leukopenia, airway hemorrhages, severe respiratory failure, and the necrotic destruction of wide areas of the lung [1]. The course of disease was fulminant, and the patient deceased on the second day of hospitalization on the intensive care unit. Molecular evaluation revealed that the isolate belonged to Multi-Locus Sequencing Type (MLST) ST152 and was positive for an extensive repertoire of virulence genes, including genes encoding for the cytotoxin Panton–Valentine leukocidine (PVL; *lukS* PV and *lukF* PV), the exotoxin *edinB*, and the recently identified exfoliative toxin E allelic variant *etE2*, identified in a case of severe necrotizing fasciitis [2].

This communication should raise awareness of the emergence of the MSSA ST152 lineage, which is associated with severe necrotizing pneumonia accompanied by bacteremia, and deep-seated soft-tissue infections as frequent presentations. In all isolates of this lineage, the newly identified exfoliative toxin gene *etE2* is present. The global dissemination of this high-risk PVL-positive community lineage, which harbors the exotoxin genes *edinB* and *etE2* implicated in the destruction of tissue integrity, is a potential threat to public health and warrants monitoring.

## 2. Case Description

A 25-year-old previously healthy male presented with tachypnea and hypoxemia to the emergency department of a regional hospital in the Netherlands in 2024. The patient had been experiencing malaise for one week prior to presentation and had been prescribed amoxicillin for a respiratory infection one day prior to admission. Upon admission, he was diagnosed with pneumosepsis and bilateral severe pneumonia, necessitating high-flow nasal cannula oxygen therapy in the intensive care unit. A nasopharyngeal swab and PCR tested positive for influenza A. Later subtyping showed that the virus belonged to H3. Blood tests revealed no leucocytosis but showed an elevated C-reactive protein level of 108 mg/L (normal range: <5 mg/L) and an arterial lactate of 6.4 mmol/L (normal range: <2 mmol/L) on admission. Given the suspicion of bacterial superinfection, empirical antibiotic therapy with cefuroxime (1.5 g three-times daily) and ciprofloxacin (400 mg twice daily) was initiated after obtaining blood and sputum cultures.

Despite treatment, the patient’s condition deteriorated rapidly, requiring endotracheal intubation and mechanical ventilation in the prone position. Hemodynamic support with escalating doses of norepinephrine and vasopressin were started, along with continuous hydrocortisone (200 mg once daily) due to the severity of septic shock.

The following day, the patient was transferred to our tertiary intensive care unit. Shortly after arrival, he suffered cardiac arrest, prompting cardiopulmonary resuscitation for approximately 20 min. During resuscitation, large amounts of pink, watery sputum were drained from the lungs. After achieving the return of spontaneous circulation, veno-venous extracorporeal membrane oxygenation and continuous renal replacement therapy were initiated. The antibiotic regimen was escalated to cefotaxime (4 g continuous daily), levofloxacin (500 mg twice daily, and clindamycin (900 mg three-times daily). Despite these interventions, the patient’s condition continued to deteriorate, with the development of liver failure, coagulopathy, refractory hyperlactatemia, heart failure, pericardial effusion, and loss of brain function, with dilated non-reactive pupils. Bronchoscopy revealed diffusely swollen bronchial mucosa and significant watery secretions. Extensive transfusions led to worsening pulmonary edema.

Given the lack of clinical stabilization, the medical team concluded that recovery was unlikely. The best supportive care was initiated, and the patient passed away later that day. *Post mortem*, the blood and sputum cultures identified *S. aureus*.

### Isolate Characterization

**Figure 1:**
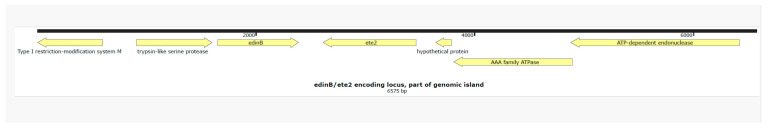
A genetic map of the part of the genomic island which integrates *etE2* and *edinB* in the chromosome of *S. aureus* UMCG-ST152-2024-1. Figure legend: The genetic map was visualized using Snapgene V 5.1.7.

**Figure 2:**
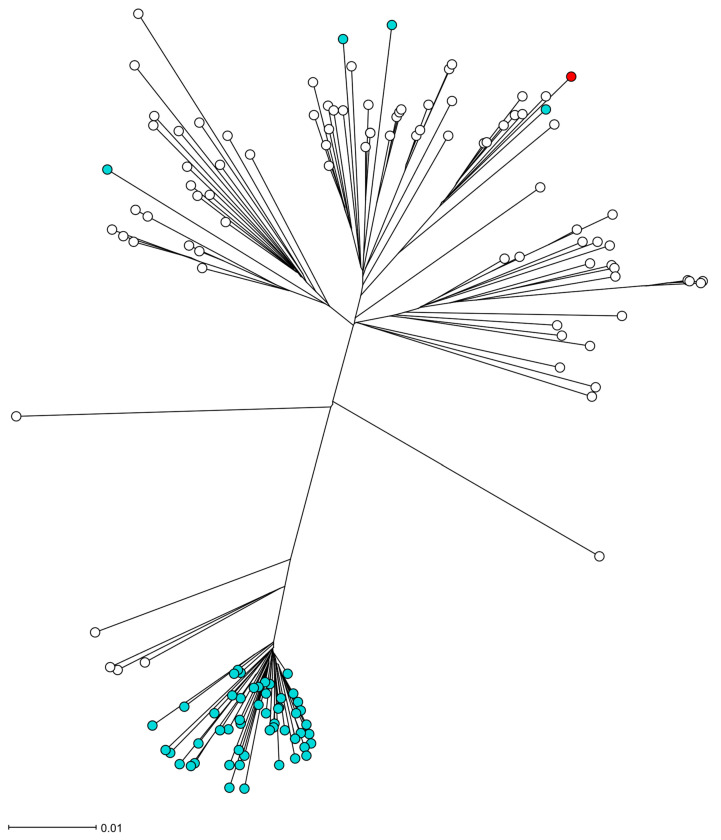
The phylogeny of the *S. aureus* isolate UMCG-ST152-2024-1 in the CC152 lineage. Figure legend: The radial neighbor-joining tree of S. aureus was generated by SeqSphere+ v9.0.8, analyzing 140 genomes. In this analysis, 1861 genes were compared using published schema. The red dot represents isolate UMCG-ST152-2024-1, the white dots are MSSA isolates, and the blue dots are MRSA isolates.

The *S. aureus* isolate UMCG-ST152-2024-1 grew from a blood culture and in pure culture from sputum samples. Analysis of short-read sequencing (MiSeq, Ilumina, San Diego, CA, USA), as described previously [3], revealed that the isolate belonged to MLST ST152. The isolate was susceptible to all antibiotics tested by Vitek2 (Biomerieux, Marcy l’Etoile, France) except for trimethoprim and penicillin G, interpreted by EUCAST v.13.1. The resistance was confirmed by agar diffusion disk testing with a zone of 6 mm to trimethoprim and 10 mm to penicillin, and the associated resistance genes *blaZ* and *dfrG* were detected by sequencing. In Table 1, we present the repertoire of virulence genes identified by SeqSphere [4]. The list of virulence factors is extensive. However, we emphasize the presence of PVL-encoding genes (*lukS* PV and *lukF* PV), *edinB*, and *etE2* since they are epidemiologically important molecular markers. Figure 1 shows that the toxin genes *etE2* and *edinB* were detected in a co-located position on a previously described genomic island [2]. The *lukF*-*PV* and *lukS-PV* genes were located on the ΦSa2 phage. In Figure 2, the phylogenetic relation of the isolate is presented using public sequencing data of isolates from clonal complex (CC) 152 [5]. The clinical isolate is in a clade that consists mostly of MSSA strains. The MSSA isolate ERR3861662 obtained from the Democratic Republic of the Congo is the most recent common ancestor of UMCG-ST152-2024-1, and it clusters with isolates that have been isolated from Nigeria (*n* = 3), Gabon (*n* = 2), Reunion, France (*n* = 2), and one isolate from Denmark which was identified as MRSA. The co-located virulence genes *etE2* and *edinB* were present in the same nucleotide composition in all isolates of the CC152 lineage.

## 3. Discussion

We present the case of a fatal community-acquired necrotizing pneumoniae caused by *S. aureus* in a young adult with underlying influenza A infection. At the University Medical Center Groningen (UMCG), severe necrotizing *S aureus* infections had sporadically been seen before. When we encounter such infections, we sequence the isolates to determine the sequence type of the *S aureus* isolate and explore if (exo-)toxins are present that could have contributed to the severity of the infection. Although disease presentation and severity of infection generally depend on combinations of virulence and host factors, some specific well-characterized virulence factors are associated with typical disease presentations.

Remarkably, when comparing the genomic data in the UMCG database, we found a closest match with a case of severe necrotizing *S. aureus* infection by MSSA ST152 presenting in 2022 [2]. This patient had been admitted to the intensive care suffering from severe necrotizing cellulitis. The isolate in that study showed a similar virulence factor profile to the present case. In that study, we reported the identification of a genomic island encoding the exotoxins *edinB* and *etE2* in the isolate’s genome. Both toxins encoded by these genes are directly associated with tissue destruction. The gene *etE2* is a novel exfoliative toxin encoding gene variant of *etE*. The *etE* gene has been detected in ovine *S. aureus* isolates, whereas we have exclusively detected *etE2* in human *S. aureus* isolates [6]. Degradation experiments of epidermal cells have shown the host-specificity of *etE*, which may explain why the toxin variants have consistently been detected in different species. Exfoliative toxins, in general, are very specific serine proteases, causing lesions in tissue by cutting desmosomes, which are cell-adherence molecules. The typical presentations of disease are bullous impetigo and staphylococcal scalded skin syndrome [7]. Future experimental studies are required to gain more insight into the contribution of the ETE2 exotoxin to the virulence of the MSSA ST152 lineage.

Our study shows that *edinB* and the co-located *etE2* are consistently present in the CC152 lineage. EDIN-B is a C3-like ADP-ribosyltransferase that catalyzes the ribosylation of Rho GTPases. The inhibition of Rho GTPases results in the modification of the actin cytoskeleton of the host cells and devastates stress fibers, which are involved in cell contractility [8]. The exposure of cells to EDIN-B results in the loss of tissue integrity through the formation of large transcellular tunnels by this exotoxin [9]. In a mouse model of *S aureus* pneumonia, the role of EDIN-B was identified as an important factor in the translocation of *S aureus* to the bloodstream by comparing wildtype and knock-out strains [10]. Also, clinical reports suggest that EDIN-B is a virulence factor associated with invasive infection. In diabetic ulcers, *edinB*-positive *S aureus* is prevalent at a much higher rate in deep-seated ulcer infections compared to low-grade infections. In addition, several studies have reported increased percentages of *edinB*-positive *S aureus* in deep-seated or bloodstream infection [11]. However, since *edinB* is co-located on a genomic island with exfoliative exotoxins, as we and others have shown [12], it is unclear if EDIN-B acts independently as a causative factor in deep-seated infections.

The PVL-positive MSSA ST152 is a hyperepidemic lineage, predominantly circulating in Africa and the Caribbean. Reports of MSSA ST152 in Europe are uncommon so far, in contrast to the MRSA clade of ST152, which is most prevalent in Europe [5]. This MRSA clade probably emerged from a common ancestor in the 1990s. This clonal expansion is similar to other hyperepidemic MRSA lineages in the community, such as CC80 and USA300. In addition, numerous sporadic acquisitions of methicillin resistance have occurred in MSSA ST152, without clonal expansion, as shown in Figure 2. The MSSA ST152 lineage is commonly associated with wound infections [13,14]. In our study, we show that MSSA ST152 is also associated with necrotizing pneumoniae.

Necrotizing pneumonia is often caused by a *S. aureus* superinfection upon a viral airway infection, most prominently influenza A and B. If the infection is caused by PVL positive isolates, the course of disease can be severe, even in young, otherwise healthy adults [1,15]. In 2022, two cases of severe necrotizing pneumonia PVL-positive MSSA ST152 requiring intensive care treatment were reported in the Indian Ocean region [16]. In both cases, an underlying SARS-CoV-2 respiratory infection had been detected, but no other immuno-compromising disease. Like our case, both the patients had concurrent bacteremia with *S. aureus*. In 2021, in a case of severe necrotizing pneumonia and bloodstream infection from the Faroe Islands, PVL-positive MSSA ST152, was reported as causative pathogen. This patient had an underlying influenza B infection, was 47 years old, and was previously healthy [17].

The pathological association between preceding influenza infection and PVL-positive *S. aureus* causing necrotizing pneumonia has already been proposed, in 2014. The proposed theory for this association is that the lungs are infiltrated by inflammatory cells during viral infection. Subsequently, PVL produced by *S. aureus* forms membrane pores, causing massive destruction of leukocytes with the subsequent release of neutrophile serine proteases that destroy human tissue [18]. While experimental data supports this theory, there are other membrane-active toxins, such as the family of small membrane-active peptides (phenol-soluble modulins, PSMs) that are pathogenetically relevant in severe soft-tissue infections, and pore-forming toxins, such as alpha-toxin, that cause the lysis of leukocytes and red blood cells [19]. Furthermore, the synergistic effect of *S. aureus* alpha-toxin Hla and PVL was shown in the pathogenesis of necrotizing pneumonia [20]. PVL could be one factor in a multifactorial etiology of disease contributed to by the numerous virulence factors that are produced by *S. aureus* implicated in the lysis of leukocytes, tissue destruction, and immune evasion. While the exact contribution to the severity of disease of each virulence factor separately is difficult to establish in humans, it is certain that the PVL-positive MSSA ST152 lineage is outstanding in its plentitude of virulence factors.

In conclusion, severe infections caused by MSSA ST152 positive for an extensive repertoire of virulence factors including PVL, *edinB*, and *etE2* appear to be an emerging issue in Europe and other regions. The presented cases and other recent casuistic studies report that isolates of this lineage cause life-threatening necrotizing pneumonia with concurrent bacteremia in previously healthy persons suffering from underlying influenza or SARS-CoV2 infections. In future surveillance projects, the typing of MSSA isolated from blood cultures could be considered for the risk assessment of this lineage. On our request, the characterized exfoliative toxin gene *etE*, which had thus far been exclusively detected in animals, and its allelic variant *etE2*, have been included in the Virulence Factor Database so that they can be detected by NGS-based analysis tools.

## Figures and Tables

**Table 1:** Major virulence genes in isolate *S. aureus* UMCG-ST152-2024-1.

Gene	Virulence Factor
aur	ACME
cap5H	capsule
cap5J	capsule
cap5K	capsule
ebpS	adhesion
edinB	exotoxin
etE2	exfoliative toxin
eno	adhesion
hla	hemolysin
hlb-intact	hemolysin
hlgA	hemolysin
hlgB	hemolysin
hlIII	hemolysin
icaA	adhesion
icaD	biofilm
isaB	immunodominant antigen
isdA	MSCRAMM
lukF-PV	leukotoxin (PVL)
lukS-PV	leukotoxin (PVL)
sak	immune-evasion
scn	immune-evasion
setB1	superantigen-like
setB3	superantigen-like
ssl01	superantigen-like
ssl02	superantigen-like
ssl10	superantigen-like
sspA	serine protease
sspB	cystein protease
sspP	cystein protease

Virulence genes were detected using SeqSphere+ v9.0.8. The exfoliative toxin *etE2* was manually detected and submitted to the Virulence Factor Database. ACME: arginine catabolic mobile element; MSCRAMM: Microbial Surface Components Recognizing Adhesive Matrix Molecules; PVL: Panton–Valentine leukocidine. Superantigen-like: A family of virulence factors with homology to superantigens, associated with host–pathogen interactions and immune evasion.

## Data Availability

The sequences of the isolates are available from the European Nucleotide Archive (ENA) under project number: PRJEB88875. [European Nucleotide Archive] URL (accessed on 15 June 2025) [https://www.ebi.ac.uk] [PRJEB88875].
